# Sequence analysis of porothramycin biosynthetic gene cluster

**DOI:** 10.1007/s12223-014-0339-x

**Published:** 2014-08-16

**Authors:** Lucie Najmanova, Dana Ulanova, Marketa Jelinkova, Zdenek Kamenik, Eliska Kettnerova, Marketa Koberska, Radek Gazak, Bojana Radojevic, Jiri Janata

**Affiliations:** 1 Institute of Microbiology AS CR, Videnska 1083, 142 20 Prague 4, Czech Republic; 2 Present address: Oceanography Section, Science Research Center, Kochi University, Kochi, Japan

## Abstract

The biosynthetic gene cluster of porothramycin, a sequence-selective DNA alkylating compound, was identified in the genome of producing strain *Streptomyces albus* subsp*. albus* (ATCC 39897) and sequentially characterized. A 39.7 kb long DNA region contains 27 putative genes, 18 of them revealing high similarity with homologous genes from biosynthetic gene cluster of closely related pyrrolobenzodiazepine (PBD) compound anthramycin. However, considering the structures of both compounds, the number of differences in the gene composition of compared biosynthetic gene clusters was unexpectedly high, indicating participation of alternative enzymes in biosynthesis of both porothramycin precursors, anthranilate, and branched L-proline derivative. Based on the sequence analysis of putative NRPS modules Por20 and Por21, we suppose that in porothramycin biosynthesis, the methylation of anthranilate unit occurs prior to the condensation reaction, while modifications of branched proline derivative, oxidation, and dimethylation of the side chain occur on already condensed PBD core. Corresponding two specific methyltransferase encoding genes *por26* and *por25* were identified in the porothramycin gene cluster. Surprisingly, also methyltransferase gene *por18* homologous to *orf19* from anthramycin biosynthesis was detected in porothramycin gene cluster even though the appropriate biosynthetic step is missing, as suggested by ultra high-performance liquid chromatography-diode array detection-mass spectrometry (UHPLC-DAD-MS) analysis of the product in the *S. albus* culture broth.

## Introduction

**Fig. 1:**
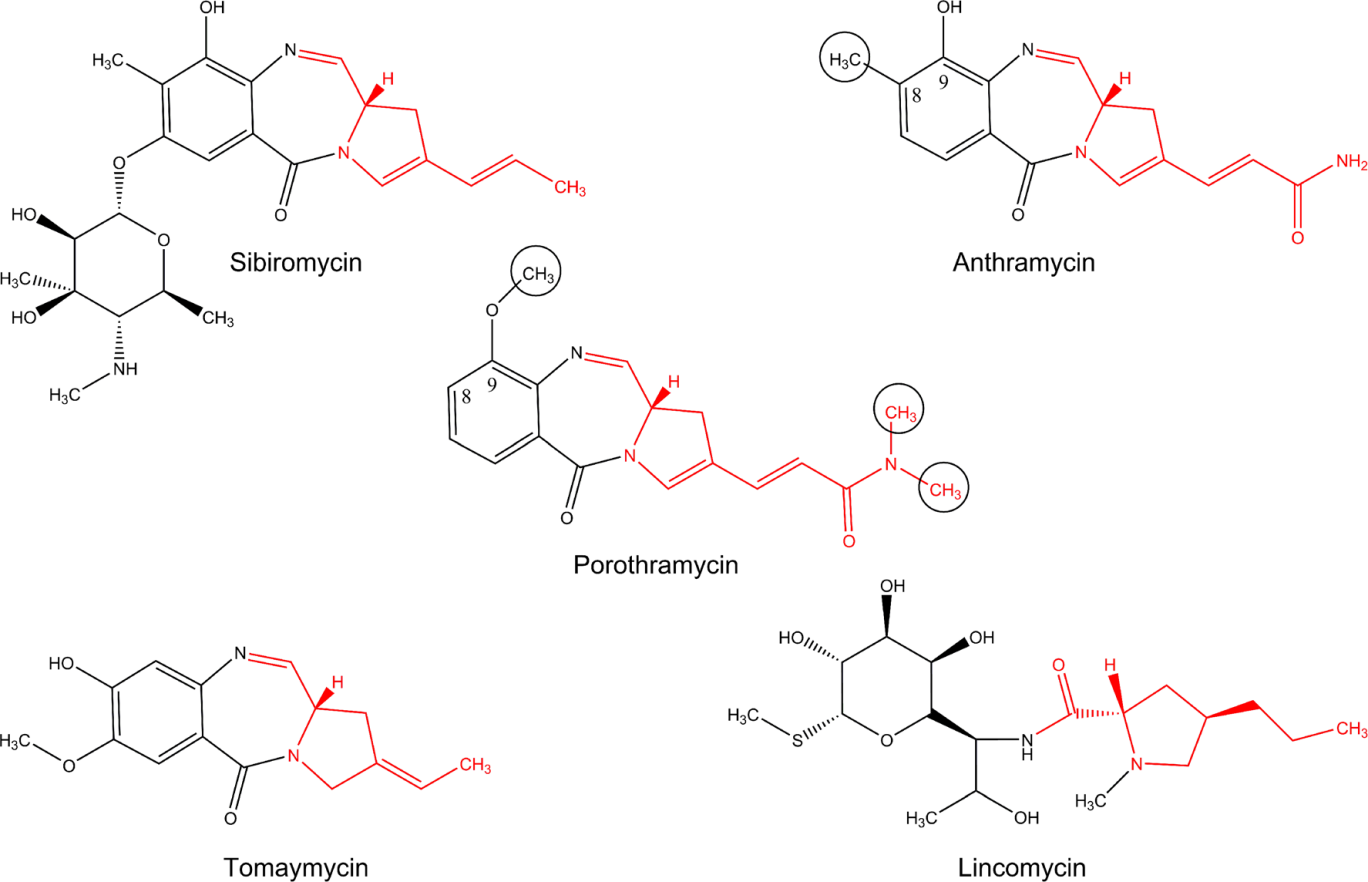
Chemical structures of porothramycin, lincomycin, and PBDs with published biosynthetic gene clusters. The branched proline derivative of a common origin is in *red*. The position modifications which differ in structures of porothramycin and its closest relative anthramycin are marked in *circles*

Pyrrolobenzodiazepines (PBDs) are sequence-selective DNA alkylating compounds exhibiting weak antimicrobial but remarkable anticancer effects. The spectrum of biological activities of the naturally produced PBDs encouraged the chemical synthesis of variety of PBDs, including dimeric and hybrid ones, resulting in improvement of the DNA-binding sequence specificity and the antineoplastic potency of this class of compounds (Gerratana 2012). However, PBDs have also a potential from another biotechnological point of view: The biosyntheses of most PBDs and commercially used antibiotic lincomycin have a common precursor, the unusual branched proline derivative (red in Fig. 1) synthesized from L-tyrosine (Brahme et al. 1984). Among lincomycin derivatives, both the length and modification of the alkyl side chain of proline moiety remarkably affect the antimicrobial activity (Magerlein 1977). The preparation of such more efficient lincomycin derivatives by mutasynthesis has been already described (Ulanova et al. 2010). Thus, an appropriate combination of biosynthetic gene clusters of PBDs and lincomycin can yield producing strains of hybrid lincosamide derivatives with improved biological properties.

The comparison of three already sequenced PBD biosynthetic gene clusters (Hu et al. 2007; Li et al. 2009a; Li et al. 2009b) and the lincomycin one (Koberska et al. 2008) reveals five or six shared homologous genes coding for enzymes converting L-tyrosine to branched L-proline derivative. In accordance with the compound structures, the sibiromycin and especially anthramycin biosynthetic gene clusters contain additional genes encoding further modifications of the proline moiety side chain. Based on the porothramycin structure (Tsunakawa et al. 1988), its biosynthetic gene cluster is expected to contain the largest set of the proline moiety modifying genes.

According to the presence/absence of the hydroxyl group in position 3 of anthranilate moiety, two distinct biosynthetic pathways have been described among PBD-producing strains: the chorismate pathway resulting in anthranilic acid, a precursor of tomaymycin biosynthesis, and the kynurenine pathway resulting in 3-hydroxyanthranilic acid, incorporated by appropriate NRPS into sibiromycin or anthramycin structure (Li et al. 2009b). The position of the hydroxyl group in porothramycin and anthramycin is identical, assigning porothramycin among PBDs employing the kynurenine type of anthranilate moiety biosynthesis. The composition of genes in porothramycin biosynthetic gene cluster thus should resemble mostly the anthramycin one, differing just in the presence/absence of three or four methyltransferase genes.

In this paper, the porothramycin biosynthetic gene cluster was sequentially characterized, compared to earlier reported biosynthetic gene clusters of PBDs (anthramycin, sibiromycin, and tomaymycin) and the unexpected gene composition is discussed.

## Materials and methods

### Genomic library construction and screening

Chromosomal DNA was isolated from *Streptomyces albus* subsp*. albus* (ATCC 39897) mycelia grown on orbital shaker in yeast extract-malt extract (YEME) broth (50 mL in a 250-mL flask) containing 0.5 % glycine, according to method described by Hopwood et al. (1985) and modified by Vachalova et al. (1995). The DNA was partially digested with BamHI (New England Biolabs) to yield DNA fragments 40–60 kbp and ligated into SuperCos I cosmid vector (Stratagene). The cosmid vector was previously linearized with XbaI (New England Biolabs), treated with calf intestine alkaline phosphatase and digested with BamHI (both New England Biolabs). The resulting ligation mixture was packaged into the λ phage, followed by phage transfection into *Escherichia coli* XL1-Blue MR^b^ strain by using protocols of Gigapack III XL Packaging Kit (Stratagene). Isolation of the cosmids was carried out on NucleoBond BAC 100 columns (Macherey-Nagel).

The *S. albus* cosmid library was screened by colony PCR using a pair of degenerate primers P1For/P1Rev (Table 1), designed based on the sequence comparison of the anthramycin, sibiromycin, and tomaymycin gene clusters (GenBank accession numbers EU195114, FJ768674, and FJ768957). The primers resemble the conserved sequence of genes encoding NRPS and kynurenine-3-monooxygenase, respectively. The cosmid CP4 from a positive clone contained 10 open reading frames (ORFs) exhibiting high similarity to already described PBD genes. However, the ORF sequence homologous to *orf7* from anthramycin biosynthesis was clearly truncated. Another pair of primers P2For/P2Rev designed according to the sequence of truncated *orf7* homolog was used for additional *S. albus* library screening to isolate cosmid CP16 harboring the remaining part of the porothramycin gene cluster.

**Table 1:** Primers for *S. albus* cosmid library screening

Name	Sequence
P1For	GCSCACATCAACTTC
P1Rev	GTTGATSCCCTGSCCGAAGAA
P2For	TGTGAGGAGGGCGTCGGGG
P2Rev	ACATCACCACCAACGAGCCC

S = G or C

### DNA sequencing and analysis

The cosmid DNA was sequenced at the DNA Sequencing Facility, University of Cambridge, UK, by a shotgun approach and subsequent primer walking to cover the gaps. The CP4 and CP16 sequences were assembled into contig using Geneious 5.3.4. software (Biomatters). The putative genes were predicted using SoftBerry online tool (www.softberry.com) and edited based on homology with already sequenced PBD gene clusters or closely related genes searched by BLAST (http://blast.ncbi.nlm.nih.gov/Blast.cgi). The BlastX was used also for prediction of putative functions of encoded proteins. The gene cluster localization in the chromosome was assessed using the BioCyc collection of pathway/genome databases at http://biocyc.org/. Nonribosomal codes were estimated by NRPSpredictor 2 at http://nrps.informatik.uni-tuebingen.de/ (Rottig et al. 2011).

### Nucleotide sequence accession number

The sequence of the porothramycin gene cluster has been deposited in GenBank under accession number HQ872605.

### Production of porothramycin

Spores of *S. albus* subsp. *albus* (ATCC 39897) were inoculated in YEME broth (50 mL) and cultivated in an orbital shaker for 24 h at 28 °C. Then, 50 mL of fresh AVM broth (yeast extract 2 g/L, (NH_4_)_2_SO_4_ 2 g/L, CaCO_3_ 5 g/L, NaCl 2 g/L, K_2_HPO_4_ 0.5 g/L, FeSO_4_ 7H_2_O 0.05 g/L, ZnSO_4_ 7H_2_O 0.05 g/L, MnSO_4_ 7H_2_O 0.05 g/L, MgSO_4_ 7H_2_O 0.1 g/L, glucose 30 g/L) was inoculated with 5 % of the pre-culture and cultivation continued for 240 h at 28 °C. Cells were harvested by centrifugation for 10 min at 4 °C, and the supernatant was used for solid-phase extraction.

### Solid-phase extraction (SPE)

Oasis HLB 3 cc 60 mg SPE cartridge (Waters, USA) was conditioned with 3 mL methanol, equilibrated with 3 mL water, and then, 3 mL cultivation broth was loaded. Subsequently, the cartridge was washed with 3 mL water, and absorbed substances were eluted with 1.5 mL methanol. The eluent was evaporated to dryness, reconstituted in 150 μL 50 % methanol, and centrifuged at 13,000 rpm for 5 min. The crude extract was analyzed by ultra high-performance liquid chromatography (UHPLC) with diode array detection (DAD) detection. For mass spectrometry (MS) detection, the crude extract was diluted 10× with 50 % methanol.

### UHPLC-DAD-MS analysis

The crude extract was analyzed on Acquity UPLC system with LCT premier XE time of flight MS analyzer (Waters). The UHPLC column Acquity UPLC BEH C_18_ (50 mm × 2.1 mm I.D., particle size 1.7 μm, Waters) and a two-component mobile phase were used for the separation. The mobile phase parts A and B consisted of 0.1 % formic acid (99 %, Merck, Germany) in water and 0.1 % formic acid in methanol (99.95 %, Chromapur GG, Chromservis, Czech Republic), respectively. The analyses were performed using a linear gradient program (min/%B) 0/5, 1.5/5, 15/90 followed by a 2-min column clean up (99 % B) and a 2-min equilibration (5 % B). The total analysis time was 19 min. The column temperature was set at 40 °C, flow rate at 0.4 mL/min, and the injection volume was 5 μL. The DAD detector acquired data from 210 to 600 nm with data sample rate 20 points per second and filter constant 0.5. The MS detector operated in the “W” positive mode with capillary voltage set at 2,800 V; cone voltage, 40 V; desolvation gas temperature, 350 °C; ion source block temperature, 120 °C; cone gas flow, 50 L/h; desolvation gas flow, 800 L/h; ion guide 1 and 2 RFs, 200 and 300 V, respectively; and hexapole RF, 150 V. The aperture 1 value was set at 0 V or at 50 V when the fragmentation by collisionally induced dissociation (CID) was carried out. The signal was acquired with the scan time, 0.1 s; interscan delay was 0.01 s (0.1 s for lock spray). The mass accuracy was kept below 5 ppm using lock spray technology with leucine enkephalin as the reference compound (2 ng/μL, 5 μL/min). The data were processed by MassLynx V4.1 software (Waters). The chromatograms were extracted at the *m*/*z* corresponding to the analyte theoretical mass; the tolerance window was ±0.015 Da.

## Results

### The assignment of the porothramycin gene cluster from *S. albus* (ATCC 39897)

**Fig. 2:**
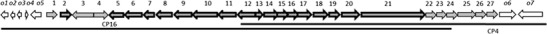
Biosynthetic gene cluster for porothramycin. The 27 *por* genes assigned to the porothramycin biosynthetic gene cluster are in *gray color* marked by respective numbers; the 18 genes homologous to the anthramycin ones are *highlighted*. The ORFs upstream *por1* and downstream *por27*, *orf1–orf7* are marked *o1–o7.* The arrangement of overlapping cosmids CP16 and CP4 is shown below

The two overlapping cosmid sequences CP4 and CP16 were assembled to give a continuous sequence of 54 kb saved in GenBank database including 39.7-kb region with 27 open reading frames (named *por1–por27*) identified and tentatively assigned to the porothramycin gene cluster by sequence analysis (Fig. 2, Table 2). The five ORFs upstream *por1* were named *orf1–5*, and another two open reading frames downstream *por27* were named *orf6* and *orf7.*

**Table 2:** ORF analysis of porothramycin biosynthetic gene cluster

Gene	No. of amino acids	Highest GenBank Homology (BlastX)	% of Identity/Similarity	Proposed Function	Anthramycin gene cluster homology (BlastX)	% of Identity/ Similarity
*por1*	177	YP_003114518	66/80	NADPH-dependent FMN reductase		
*por2*	227	AFU02188	68/77	two component transcriptional regulator	ORF25 (ABW71856)	66/77
*por3*	743	WP_020540464	75/86	ABC transporter (UvrA family)		
*por4*	316	WP_019357233	75/83	DeoR family transcription regulator		
*por5*	479	ABW71855	70/81	flavin-containing oxidoreductase	ORF24 (ABW71855)	70/81
*por6*	400	ABW71841	70/82	MFS transporter	ORF10 (ABW71841)	70/82
*por7*	258	ABW71840	61/72	hydroxylase/glyoxylase (bleomycin res)	ORF9 (ABW71840)	61/72
*por8*	622	ABW71832	72/81	amidotransferase	ORF1 (ABW71832)	72/81
*por9*	397	ABW71835	73/83	cytochrome P450 hydroxylase	ORF4 (ABW71835)	73/83
*por10*	350	ABW71836	76/86	C-Methyltransferase	ORF5 (ABW71836)	76/86
*por11*	599	ABW71837	81/87	gamma-glutamyltransferase	ORF6 (ABW71837)	81/87
*por12*	471	ABW71838	73/82	FAD oxidoreductase	ORF7 (ABW71838)	73/82
*por13*	156	ABW71843	72/83	L-DOPA 2,3-dioxygenase	ORF12 (ABW71843)	72/83
*por14*	305	ABW71844	61/70	L-Tyrosine 3-hydroxylase	ORF13 (ABW71844)	61/70
*por15*	297	ABW71845	82/88	F420 dependent reductase	ORF14 (ABW71845)	82/88
*por16*	289	ABW71846	65/71	unknown (putative isomerase)	ORF15 (ABW71846)	65/71
*por17*	415	ABW71847	75/81	kynureninase	ORF16 (ABW71847)	75/81
*por18*	347	YP_006809167	73/85	aromatic C-methyltransferase	ORF19 (ABW71850)	73/81
*por19*	288	WP_017545377	71/76	aryl formamidase	ORF20 (ABW71851)	74/80
*por20*	607	ABW71852	73/84	NRPS	ORF21 (ABW71852)	73/84
*por21*	1938	ABW71853 + ABW71854	71/80 + 72/81	NRPS + kynurenine 3-monooxygenase	ORF22 (ABW71853) + ORF23 (ABW71854)	71/80 + 72/81
*por22*	290	WP_017974834	67/77	NmrA family transcriptional regulator		
*por23*	477	WP_005249919	76/86	FMNH2-dependent monooxygenase		
*por24*	445	ABZ09841	65/75	glutamine synthetase		
*por25*	807	WP_003888341	63/76	Aminomethyltransferase		
*por26*	352	WP_010304927	69/79	O-methyltransferase		
*por27*	448	WP_005445166	73/85	Flavin-containing monooxygenase FMO		

Eighteen out of 27 *por* genes resemble significant similarity to genes from anthramycin biosynthetic gene cluster and many of them also to other PBD biosynthetic gene clusters. Remaining nine *por* genes were assigned to porothramycin biosynthesis based on the position in the gene cluster or according to the proposed function of the respective proteins.

### Estimation of borders and localization of the gene cluster in genome

Three putative ORFs upstream of *por1* (*orf1–*3) are similar to fragments of IS116/IS110/IS902 family transposases indicating probable place of gene cluster integration into the genome. Other two following putative ORFs (*orf4* and *orf5*) exhibit only partial and very low sequence similarity to transcriptional regulators, making any prediction of putative function impossible. Thus, the gene *por1*, coding for putative NADPH-dependent FMN reductase probably supporting one or two monooxygenases encoded by genes *por23* and *por27*, was assigned to be the first coding sequence of the porothramycin gene cluster. The *orf6* immediately downstream of *por27* codes for putative protein, exhibiting ~80 % identity to endoglycoceramidases, the enzymes hydrolyzing a β-glycosidic linkage between oligosaccharides and ceramides in various glycosphingolipids. In the porothramycin biosynthetic pathway, no such activity is expected indicating *por27* to be the last gene of porothramycin gene cluster.

In order to localize the porothramycin biosynthetic gene cluster position in the chromosome, the sequences of ~4,500 nt upstream of *por1* and ~7,000 nt downstream of *por27* were compared with the already sequenced genome of nonproducing strain *S. albus* J1077 (GenBank accession number CP004370.1). However, no similarity was detected, indicating that the probable position of the gene cluster is in one of the terminal arms of the chromosome. Further, this hypothesis was supported by the sequence comparison with other already sequenced streptomycete genomes using the BioCyc tools. The genes homologous to the *orf6* downstream of *por27* (coding for putative endoglycoceramidase) are, when present, localized mostly in one of the terminal arms of the chromosome (*S. avermitilis*, *S. bingchenggensis BCV-1*, *S. hygroscopicuc jingganensis 5008*, or *S. scaibei 87.82*).

### Comparative analysis of porothramycin biosynthetic gene cluster

As expected, the highest number of homologs of 27 *por* genes, 18, can be found in the anthramycin biosynthetic gene cluster. Moreover, with only three exceptions, the encoded proteins from anthramycin biosynthesis are the closest homologs of the porothramycin ones. On the other hand, the number of identified differences in the gene composition is unexpectedly high. Based on the compound structures, only the presence of 2–3 porothramycin-specific methyltransferase genes and absence of *orf19* homolog coding anthramycin-specific methyltransferase were supposed. Surprisingly, the *orf19* homolog was detected in porothramycin gene cluster (*por18*), while homologs of another six anthramycin genes (*orf2*, *orf3*, *orf8*, *orf11*, *orf17*, and *orf18*) were not. Additionally, homologs of nine porothramycin genes (*por1*, *por3*, *por4*, and *por22*–*por27*) are not present in the anthramycin gene cluster.

The absence of three anthramycin homologs in the porothramycin gene cluster can be easily explained: *orf11* and *orf18* are too short without any assigned function. The porothramycin gene cluster sequence thus indicates that the definition of these ORFs was probably artificial. The *orf8* encodes the resistance determinant of ATP-binding cassette (ABC) transporter family. The porothramycin-specific gene *por3* codes for a putative ABC transporter of UvrA family, probably exhibiting similar function to the *orf8* product.

The absence of other three anthramycin homologs reflects unexpected differences in the biosynthesis of both building units, anthranilate (*orf17*) and branched proline derivative (*orf2* and *orf3*). The *orf17* codes for tryptophan 2,3-dioxygenase (TDO) catalyzing the first reaction of kynurenine pathway leading to one of final compound precursors, 3-hydroxyanthranilic acid. The *orf2* and *orf3* genes code for dehydrogenases which are supposed to oxidize side chain of branched proline derivative precursor. We suggest that three porothramycin specific genes (*por1*, *por23*, and *por27*) could replace the missing *orf2* and *orf3* homologs in the alternative pathway leading to branched proline derivative. The proposed modifications of both precursors’ biosynthetic pathways will be discussed.

The presence of two out of five remaining porothramycin specific genes, *por25* and *por26* coding for methyltransferases, was predicted. According to BlastX analysis, the *por26* product putatively catalyzes *O*-methylation of porothramycin C-9 hydroxyl while putative aminomethyltransferase Por25 could be responsible for dimethylation of proline derivative building unit side chain. The presence of remaining two putative regulatory genes (*por4* and *por22*) and putative glutamine synthetase gene (*por24*) was not expected but could be consistent with the proposed biosynthetic pathway and will be discussed.

Two putative NRPS coding genes *por20* and *por21* were identified. The *por20* is homologous to NRPS coding gene *orf21* from anthramycin biosynthesis responsible for anthranilate building unit activation. According to BlastX analysis, the 5,817 nt long *por21* seems to be a fusion gene coding for NRPS and kynurenine 3-monooxygenase. The respective NRPS part is homologous to ORF22 from anthramycin biosynthesis, which is responsible for recognition and activation of proline derivative building block and condensation reaction. Respective kynurenine 3-monooxygenase is homologous to ORF23, catalyzing the third reaction of kynurenine pathway.

The NRPS adenylation domain (A-domain) activates the NRPS substrate for further condensation. The A-domain substrate binding pocket is formed by a set of 10 amino acid residues, often called the “nonribosomal code”, which side chains contact the substrate (Conti et al. 1997; Stachelhaus et al. 1999) and thus determine the substrate specificity of A-domains. Analysis of the nonribosomal code of an uncharacterized A-domain can thus often predict its substrate specificity. Comparison of both Por20 and Por21 nonribosomal codes with their respective homologs from anthramycin and sibiromycin biosynthesis is shown in Table 3. According to BlastX analysis of full-length NRPS modules, Por20 was found to be the closest relative of ORF21 with significantly higher homology when compared to SibE from sibiromycin biosynthesis (Table 3(A)). However, when comparing the nonribosomal codes only, ORF21 and SibE are identical, whereas Por20 differs in three amino acid residues, indicating that the recognized anthranilate precursor of porothramycin probably differs from that of anthramycin and sibiromycin. On the other hand, only a single difference between nonribosomal codes of Por21 and ORF22 indicates identical or nearly identical proline derivative precursors in biosyntheses of porothramycin and anthramycin. These outputs enable to predict the substrates of condensation reaction as discussed later.

**Table 3:**
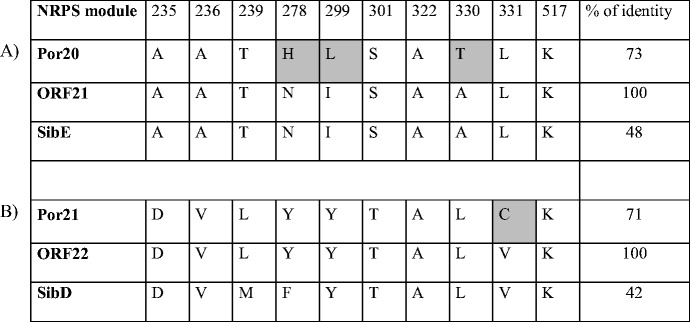
Comparison of nonribosomal codes and the overall identity of A-domains activating derivatives of anthranilate (A) and branched L-proline (B) involved in biosynthesis of PBDs

Amino acid positions are numbered at the top according to the PheA, A-domain of GrsA (Conti et al. 1997). Amino acid residues in porothramycin A-domains differing from anthramycin ones are marked in *gray*. The percentage of overall NRPS module identity to anthramycin NRPS modules (ORF21, ORF22) was estimated by BlastX analysis.

### UHPLC-DAD-MS analysis of porothramycin

**Fig. 3:**
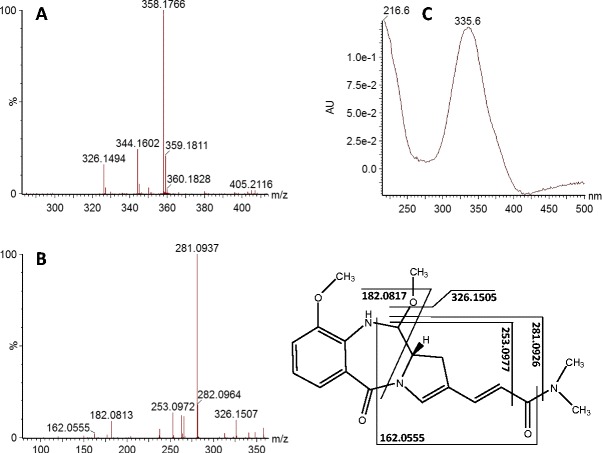
UHPLC-DAD-MS analysis of *S. albus* culture broth reveals porothramycin. **a** Mass spectrum of the porothramycin methoxy form, **b** CID mass spectrum (fragmentation pattern), **c** UV spectrum

The culture broth of *S. albus* was purified by solid-phase extraction, and the crude extract was analyzed by UHPLC-DAD-MS. The analysis revealed an ion-extracted chromatographic peak of the porothramycin methoxy form at the retention time of 7.49 min. The corresponding mass spectrum (Fig. 3a) shows the major peak of 358.1766, which is in accordance with the theoretical value of 358.1768 for the [M + H]^+^ adduct of porothramycin methoxy form. These data were further supported by the fragmentation pattern in the CID mass spectrum (Fig. 3b). In addition, the UV spectrum of this compound exhibited a maximum at 335 nm (Fig. 3c), which complies with data that have been reported for porothramycin (Tsunakawa et al. 1988). To sum up, the high resolution mass spectrometry data, including the fragmentation pattern of CID mass spectrum and the UV spectrum provide very convincing evidence that the culture broth of *S. albus* contained the porothramycin.

### Porothramycin forms

All PBDs including porothramycin can be found in three different forms, depending on storage or analysis conditions. The imine and carbinolamine forms are in equilibrium in aqueous solutions, whereas the methoxy form is formed in methanol and is in equilibrium with the carbinolamine form (Barkley et al. 1991; Petrusek et al. 1981; Hurley et al. 1988; Remers et al. 1986; Leimgrub et al. 1965). We have detected the porothramycin by MS as the porothramycin methoxy form (see the chemical structure in Fig. 3b) due to the presence of methanol in the mobile phase. Since this methoxy group at C11 is not relevant in the context of porothramycin biosynthesis, we consider the imine form (see Fig. 1) to be the parent compound as has been recommended (Gerratana 2012).

## Discussion

### The assignment of the analyzed gene cluster to porothramycin biosynthesis

Prior to discussion of individual gene functions, the assignment of the whole gene cluster to the final compound, porothramycin, should be solved. The primers for analysis of *S. albus* chromosomal DNA and cosmid library were designed based on the sequences of ORF14, ORF22, and ORF23 from anthramycin biosynthesis and respective homologous genes from sibiromycin, and in case of ORF14 also tomaymycin and lincomycin biosyntheses. Even so, no additional sequences except for those corresponding to the analyzed and published gene cluster HQ872605 were identified. The analysis of *S. albus* subsp. *albus* (ATCC 39897) cultivation media accordingly revealed the single main product structurally corresponding to porothramycin and several minor products corresponding to predicted porothramycin biosynthesis pathway if one or two methylation steps were omitted. Thinking about hypothetical presence of other PBD coding gene cluster in *S. albus* subsp. *albus* (ATCC 39897), it is important to note that the genome of taxon belonging to the identical species, *S. albus* J1074, was already fully sequenced. Although its five putative biosynthetic gene clusters contain one or multiple NRPS coding genes, none of them exhibit similarity to any PBD coding gene.

Regardless the clear evidence based on the gene inactivation is absent, the above-mentioned data together provide indirect but fairly strong evidence that the presence of additional PBD biosynthetic gene cluster is highly improbable in the *S. albus* subsp. *albus* (ATCC 39897) genome.

### Biosynthesis of 3-hydroxyanthranilic acid

**Fig. 4:**
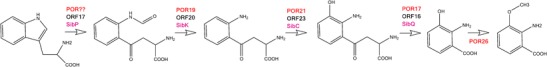
Proposed biosynthetic pathway for anthranilate moiety. Appropriate enzymes are marked: POR for porothramycin, Sib for sibiromycin, and ORF for anthramycin biosynthesis

In anthramycin and sibiromycin biosynthesis, the 3-hydroxyanthranilic acid results from kynurenine pathway (Fig. 4) catalyzed by homologous sets of four enzymes (Li et al. 2009b). Porothramycin gene cluster encodes three of them indicating the expected use of kynurenine pathway. However, the gene homologous to *orf17*/*sibP* encoding TDO catalyzing initial step of the pathway is missing. As the kynurenine pathway in primary metabolism represents a major route of tryptophan catabolism, even for streptomycetes (Zummo et al. 2012; Kurnasov et al. 2003), one can expect the use of primary metabolic TDO for this reaction. The possible employment of a primary metabolic TDO in the secondary metabolite biosynthesis was described for daptomycin biosynthesis where the residual antibiotic production was observed even after deletion of appropriate TDO encoding the cluster gene (Baltz et al. 2010). The residual antibiotic production was explained by the expression of alternative, primary metabolic TDO. In *S. albus* J1074 genome, the primary metabolism TDO coding gene was identified (GenBank accession number CP004370.1). The relevant protein in producing strain *S. albus* subsp*. albus* (ATCC 39897) could thus serve this function in porothramycin biosynthesis.

### Dehydroproline acryl-(*N*′,*N* ′-dimethyl)amide biosynthesis

**Fig. 5:**
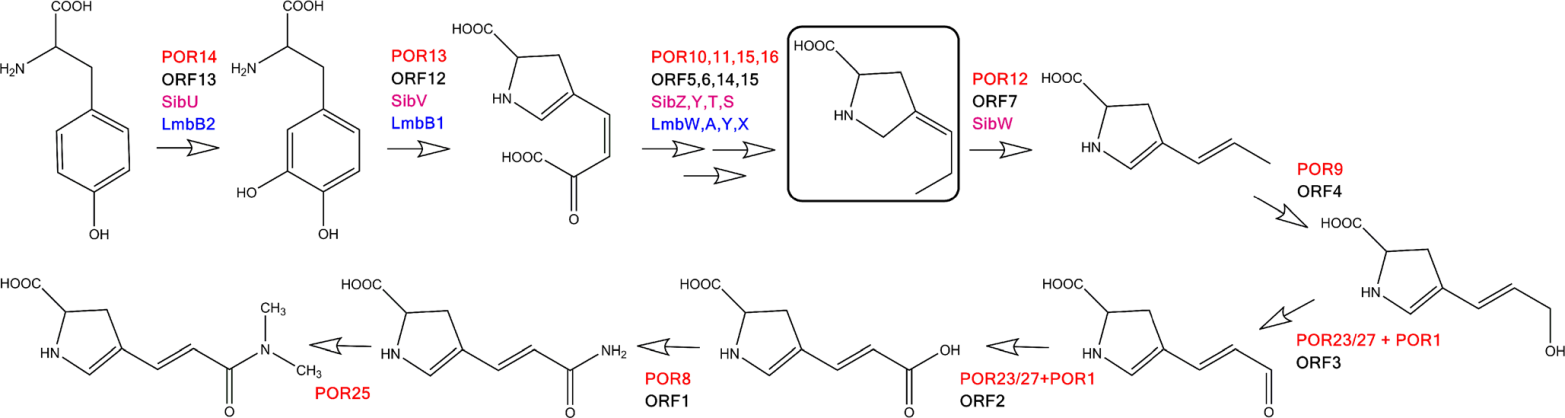
Proposed biosynthetic pathway of dehydroproline acryl-(*N′*,*N′*-dimethyl)amide moiety. The compound in frame is supposed to be the subject of condensation reaction. The following side chain modifications thus occur probably already on the condensed PBD core structure. Appropriate enzymes are marked: POR for porothramycin, Sib for sibiromycin, Lmb for lincomycin, and ORF for anthramycin biosynthesis

The branched proline derivative moiety of porothramycin differs from that of anthramycin only in the additional dimethylation on the amide group of the side chain. Besides the specific methyltransferase gene (*por25*, discussed later), the set of biosynthetic genes should fully resemble the anthramycin one. Accordingly, in the porothramycin biosynthetic gene cluster, there was identified a full set of six genes (*por10*, *por11*, and *por13* to *por16*) with high-sequence similarity not only to anthramycin biosynthetic genes (Table 2) but also to six genes involved in biosynthesis of 4-propyl-L-proline precursor of lincomycin (GenBank accession number EU124663, Koberska et al. 2008) and genes assigned to branched proline derivative biosynthesis from sibiromycin and tomaymycin gene clusters (Fig. 5). The similarity is not surprising considering already described common origin of branched proline derivative precursors in both groups of natural compounds (Hu et al. 2007). In accordance with the structural similarity of porothramycin and anthramycin, also *por12* coding for a putative FAD oxidoreductase has its counterpart in the anthramycin biosynthesis (*orf7*).

However, considering the remaining four anthramycin genes (*orf1–4*) coding for putative amidotransferase (*orf1*), aldehyde dehydrogenase (*orf2*), alcohol dehydrogenase (*orf3*), and cytochrome P450 hydroxylase (*orf4*), respectively, only *orf1* and *orf4* have their homologs in the porothramycin biosynthesis (*por8* and *por9*). We suppose that the candidate genes coding for alternative enzymes catalyzing the two missing oxidation steps in the proposed biosynthetic pathway could be *por23* and *por27*, specific for porothramycin gene cluster only*.* The product of gene *por23* exhibits 76 % identity to putative FMNH2-dependent monooxygenase from *Rhodococcus wratislaviensis* (WP_005567976), and product of *por27* is 73 % identical to a putative flavin-containing monooxygenase from *Saccharomonospora azurea* (WP_005445166). Both monooxygenase genes are highly conserved among bacteria but are not frequently found in streptomycetes.

According to BlastX analysis, the gene *por1* exhibits high similarity to NADPH-dependent FMN reductases. Many FMN-dependent two-component monooxygenase systems, consisting of NADPH-dependent FMN reductase and FMNH2-dependent monooxygenase, have been described, capable of catalyzing variety of different reactions including oxidation of aromatic and polycyclic compounds or long-chain alkanes (for review see Ellis 2010). We thus hypothesize the product of *por1* to provide a reduced FMN for FMNH2-dependent monooxygenase encoded by *por23* and/or *por27*.

We realize that without inactivation of appropriate genes in the porothramycin producing strain, the proposed alternative mechanism of branched proline derivative side-chain oxidation remains speculative. The employment of oxidoreductase(s) out of the gene cluster cannot be excluded. Another speculative role of the Por1 + Por23 + Por27 protein set could be the function equivalent to missing TDO (homologous to ORF17 from anthramycin biosynthesis), i.e. an oxidative ring opening of tryptophan, the first biosynthetic step of anthranilate moiety biosynthesis. Finally, regarding positions of *por1* and *por27* just at the estimated borders of the gene cluster, we cannot exclude also the possibility that those genes are not involved in the porothramycin biosynthesis at all.

### Methyltransferase genes

The only difference between porothramycin and anthramycin structures is in the modification of core structure by methyl groups (Fig. 1). Accordingly, in the porothramycin gene cluster, two specific methyltransferase genes were identified. The gene *por25* codes for a protein exhibiting high similarity to aminomethyltransferases. We propose that its product is responsible for the dimethylation of the amide group of the branched proline derivative moiety. The gene *por26* codes for putative *O*-methyltransferase, which possibly methylates the C-9 hydroxyl of porothramycin.

The presence of the third methyltransferase gene *por18* was not expected and cannot be easily explained. Por18 is homologous to ORF19 and SibL from anthramycin and sibiromycin gene cluster, respectively which are proposed to methylate the C-8 position of aromatic ring of appropriate PBD. However, the methylation of C-9 hydroxyl and not C-8 of aromatic ring was previously demonstrated in the structure of porothramycin (Tsunakawa et al. 1988). In this paper, we confirmed by UHPLC-DAD-MS analysis of *S. albus* culture broth the production of PBD compound with characteristics corresponding to porothramycin (Fig. 3). The fragmentation pattern corresponds to the compound with only one methylation on the anthranilate moiety. Even though the analysis does not allow determining which particular position of the moiety is methylated, we can exclude the methylation of both the positions, C-9 hydroxyl and C-8, at once. Summarized, the Por18 methylation activity seems not to be employed in porothramycin biosynthesis. The sequence analysis of *por18* locus did not reveal any apparent reason: The ORF is complete; the putative protein product exhibits high similarity to ORF19 and SibL; and the gene has a fair Shine-Dalgarno sequence. The regulation of gene expression could possibly explain this missing activity.

### Prediction of condensation reaction substrates

It is in question, if the authentic substrate of the *por12* is the compound in frame in Fig. 5, or rather already condensed PBD core structure, i.e. which branched proline derivative precursor is condensed with the anthranilate building unit. According to high conservation of nonribosomal codes of Por21/ORF22/SibD activating appropriate proline derivative precursors, the activation of identical or highly similar substrates seems to be more probable. Thus, we suppose the condensation step to occur early in the biosynthesis, probably before Por12 action. The further pathway-specific modifications of proline moiety probably take part on the condensed PBD core structure. On the other hand, three differences were found between nonribosomal codes of otherwise closely related Por20 and ORF21. Analogical situation was described in biosynthesis of lincosamides, lincomycin, and celesticetin (Kadlcik et al. 2013). Respective A-domains LmbC and CcbC exhibit the highest overall mutual homology but radically differ in the nonribosomal codes. Whereas L-proline activating CcbC fulfills the L-proline consensus, LmbC that was proved to activate 4-propyl-L-proline differs in five amino acid residues. In the case of PBD biosynthesis, the difference of Por20 from both ORF21/SibE nonribosomal codes indicates that anthranilate precursor in porothramycin biosynthesis is not identical with the antramycin/sibiromycin biosynthetic pathway. As anthranilate moieties are differently modified in final compounds (methylation of C-8 in anthramycin and sibiromycin vs methylation of hydroxyl at C-9 in porothramycin), we predict that the respective anthranilate precursors enter the condensation already appropriately modified. Summarized we suppose that in porothramycin biosynthesis, the methylation of anthranilate unit catalyzed by Por26 occurs prior the condensation reaction, while modifications of branched proline derivative starting probably with a reaction catalyzed by Por12 occurs on already condensed PBD core.

### Other porothramycin-specific genes

The BlastX analysis of the porothramycin gene cluster revealed also three other porothramycin-specific genes: two putative regulatory genes *por4* and *por22* and *por24* encoding putative glutamine synthetase. Glutamine synthetases are ubiquitous enzymes essential for nitrogen metabolism catalyzing the condensation of ammonium and glutamate to form glutamine. Among others, they participate also in the biosynthesis of L-tryptophan, the starting compound of 3-hydroxyanthranilic acid biosynthesis. The product of *por24* thus could possibly act to enhance the intracellular level of L-tryptophane, available for porothramycin biosynthesis. Moreover, *por22* is a putative regulatory gene, in which product resembles the highest similarity to NmrA family proteins, known to participate in controlling nitrogen metabolism (Andrianopoulos et al. 1998). The putative product of remaining gene *por4* exhibits the highest sequence similarity to DeoR family transcriptional regulators.

## References

[CR1] Andrianopoulos A, Kourambas S, Sharp JA, Davis MA, Hynes MJ (1998). Characterization of the *Aspergillus nidulans nmrA* gene involved in nitrogen metabolite repression. J Bacteriol.

[CR2] Baltz RH, Nguyen KT, Alexander DC (2010) Reprogramming daptomycin and A54145 biosynthesis to produce novel lipopeptide antibiotics. In: Wu-Kuang Yeh H-CY, James R. McCarthy (ed) Enzyme Technologies: Metagenomics, Evolution, Biocatalysis, and Biosynthesis. John Wiley & Sons, Inc., pp 285-307. doi:10.1002/9780470627303.ch9

[CR3] Barkley MD, Thomas TJ, Maskos K, Remers WA (1991). Steady-state fluorescence and molecular-modelling studies of tomaymycin DNA adducts. Biochemistry.

[CR4] Brahme NM, Gonzalez J, Mizsak S, Rolls J, Hessler E, Hurley L (1984). Biosynthesis of the lincomycins. 2. Studies using stable isotopes on the biosynthesis of methylthiolincosaminide moiety of lincomycin A. J Am Chem Soc.

[CR5] Conti E, Stachelhaus T, Marahiel MA, Brick P (1997). Structural basis for the activation of phenylalanine in the non-ribosomal biosynthesis of gramicidin S. EMBO J.

[CR6] Ellis HR (2010). The FMN-dependent two-component monooxygenase systems. Arch Biochem Biophys.

[CR7] Gerratana B (2012). Biosynthesis, synthesis, and biological activities of pyrrolobenzodiazepines. Med Res Rev.

[CR8] Hopwood DA, Bibb M, Chater K, Kieser T, Bruton CJ, Kieser HM, Lydiate DJ, Smith CP, Ward JM, Schrempf H (1985) Genetic manipulation of *Streptomyces.* A Laboratory Manual. Norwich

[CR9] Hu Y, Phelan V, Ntai I, Farnet CM, Zazopoulos E, Bachmann BO (2007). Benzodiazepine biosynthesis in *Streptomyces refuineus*. Chem Biol.

[CR10] Hurley LH, Reck T, Thurston DE, Langley DR, Holden KG, Hertzberg RP, Hoover JRE, Gallagher G, Faucette LF, Mong SM, Johnson RK (1988). Pyrrolo 1,4 benzodiazepine antitumor antibiotics—relationship of DNA alkylation and sequence specificity to the biological activity of natural and synthetic compounds. Chem Res Toxicol.

[CR11] Kadlcik S, Kucera T, Chalupska D, Gazak R, Koberska M, Ulanova D, Kopecky J, Kutejova E, Najmanova L, Janata J (2013). Adaptation of an L-proline adenylation domain to use 4-propyl-L-proline in the evolution of lincosamide biosynthesis. PLoS ONE.

[CR12] Koberska M, Kopecky J, Olsovska J, Jelinkova M, Ulanova D, Man P, Flieger M, Janata J (2008). Sequence analysis and heterologous expression of the lincomycin Biosynthetic cluster of the type strain *Streptomyces lincolnensis* ATCC 25466. Folia Microbiol.

[CR13] Kurnasov O, Goral V, Colabroy K, Gerdes S, Anantha S, Osterman A, Begley TP (2003). NAD biosynthesis: Identification of the tryptophan to quinolinate pathway in bacteria. Chem Biol.

[CR14] Leimgrub W, Stefanov V, Schenker F, Karr A, Berger J (1965). Isolation and characterization of anthramycin a new antitumor antibiotic. J Am Chem Soc.

[CR15] Li W, Chou SC, Khullar A, Gerratana B (2009). Cloning and characterization of the biosynthetic gene cluster for tomaymycin, an SJG-136 monomeric analog. Appl Environ Microbiol.

[CR16] Li W, Khullar A, Chou S, Sacramo A, Gerratana B (2009). Biosynthesis of sibiromycin, a potent antitumor antibiotic. Appl Environ Microbiol.

[CR17] Magerlein BJ, Pearlman D (1977). Modification of lincomycin. Structure-activity relationships among the semisynthetic antibiotics.

[CR18] Petrusek RL, Anderson GL, Garner TF, Fannin QL, Kaplan DJ, Zimmer SG, Hurley LH (1981). Pyrrolo 1,4 benzodiazepine antibiotics—proposed structures and characteristics of the in vitro deoxyribonucleic-acid adducts of anthramycin, tomaymycin, sibiromycin and neothramycin-A and neothramycin-B. Biochemistry.

[CR19] Remers WA, Mabilia M, Hopfinger AJ (1986). Conformations of complexes between pyrrolo 1,4 benzodiazepines and DNA segments. J Med Chem.

[CR20] Rottig M, Medema MH, Blin K, Weber T, Rausch C, Kohlbacher O (2011). NRPSpredictor2—a web server for predicting NRPS adenylation domain specificity. Nucleic Acids Res.

[CR21] Stachelhaus T, Mootz HD, Marahiel MA (1999). The specificity-conferring code of adenylation domains in nonribosomal peptide synthetases. Chem Biol.

[CR22] Tsunakawa M, Kamei H, Konishi M, Miyaki T, Oki T, Kawaguchi H (1988). Porothramycin, a new antibiotic of the anthramycin group—production, isolation, structure and biological activity. J Antibiot.

[CR23] Ulanova D, Novotna J, Smutna Y, Kamenik Z, Gazak R, Sulc M, Sedmera P, Kadlcik S, Plhackova K, Janata J (2010). Mutasynthesis of lincomycin derivatives with activity against drug-resistant staphylococci. Antimicrob Agents Chemother.

[CR24] Vachalova K, Felsberg J, Petricek M, Spizek J, Tichy P (1995). Copy number determination of different derivatives of the streptomycete mini-plasmid pSLG33. Folia Microbiol.

[CR25] Zummo FP, Marineo S, Pace A, Civiletti F, Giardina A, Puglia AM (2012). Tryptophan catabolism via kynurenine production in *Streptomyces coelicolor*: identification of three genes coding for the enzymes of tryptophan to anthranilate pathway. Appl Microbiol Biotechnol.

